# A key process controlling the wet removal of aerosols: new observational evidence

**DOI:** 10.1038/srep34113

**Published:** 2016-10-05

**Authors:** Sho Ohata, Nobuhiro Moteki, Tatsuhiro Mori, Makoto Koike, Yutaka Kondo

**Affiliations:** 1 Department of Earth and Planetary Science, Graduate School of Science, The University of Tokyo, Tokyo, Japan; 2 National Institute of Polar Research, Tokyo, Japan

## Abstract

The lifetime and spatial distributions of accumulation-mode aerosols in a size range of approximately 0.05–1 μm, and thus their global and regional climate impacts, are primarily constrained by their removal via cloud and precipitation (wet removal). However, the microphysical process that predominantly controls the removal efficiency remains unidentified because of observational difficulties. Here, we demonstrate that the activation of aerosols to cloud droplets (nucleation scavenging) predominantly controls the wet removal efficiency of accumulation-mode aerosols, using water-insoluble black carbon as an observable particle tracer during the removal process. From simultaneous ground-based observations of black carbon in air (prior to removal) and in rainwater (after removal) in Tokyo, Japan, we found that the wet removal efficiency depends strongly on particle size, and the size dependence can be explained quantitatively by the observed size-dependent cloud-nucleating ability. Furthermore, our observational method provides an estimate of the effective supersaturation of water vapour in precipitating cloud clusters, a key parameter controlling nucleation scavenging. These novel data firmly indicate the importance of quantitative numerical simulations of the nucleation scavenging process to improve the model’s ability to predict the atmospheric aerosol burden and the resultant climate forcings, and enable a new validation of such simulations.

Atmospheric aerosols in the accumulation-mode size range of approximately 0.05–1 μm greatly affect Earth’s radiation budget directly by scattering and absorbing sunlight and indirectly by modifying the properties of clouds^1^,^2^. The key factors that affect these climate impacts, including the number and mass concentrations, lifetimes, vertical distributions, and long-range transport of accumulation-mode aerosols, are commonly regulated by the removal process associated with cloud and precipitation (wet removal)^3^,^4^. Therefore, a fundamental understanding and quantitative numerical modelling of the wet removal process are critical for evaluating aerosol’s climate forcing, which is the major scientific uncertainty in predicting human-induced climate change in this century^5^,^6^.

Incorporation of an aerosol particle into a water droplet (scavenging) in a cloud-and-precipitation system can occur via two distinct physical mechanisms: nucleation scavenging and impaction scavenging^3^ (see Fig. 1). The former mechanism refers to an aerosol (cloud condensation nuclei: CCN) inducing formation of a cloud droplet in supersaturated water vapour, and the latter mechanism refers to collision-coalescence of an aerosol and a water droplet. Scavenged aerosols are irreversibly removed from the atmosphere if the water droplets gravitationally fall and reach the ground.

According to theoretical studies using numerical models, the number concentration of accumulation-mode aerosols can be reduced by up to 94% by nucleation scavenging^7^,^8^. However, the relative importance of the nucleation and impaction scavenging mechanisms on wet removal in actual precipitating clouds remains unknown because of the lack of observational methods. Although a comparison of the chemical compositions of filter-sampled aerosols and collected rainwater indicated a higher wet removal efficiency of more hygroscopic aerosol compounds^9^,^10^, such a bulk chemical analysis is unable to confirm the detailed relationships between the particle size and wet removal of aerosols. Recent aircraft observations found that the size of black carbon aerosols were smaller in an air mass that had experienced a greater amount of precipitation, implying a substantial particle-size dependence of wet removal^11^,^12^. Despite these observational implications of causal links between the microphysical properties of aerosols and their wet removal efficiency, no quantitative data have been available for identifying the critical mechanism of wet removal.

In this study, we introduce a novel observational method to reveal the relative contribution of the nucleation scavenging mechanism compared with the impaction scavenging mechanism in precipitating clouds, using black carbon (BC) aerosol as an observable particle tracer during the removal process. The BC-tracer method is based on simultaneous ground-based observations of BC in the planetary boundary layer air (prior to removal) and in rainwater (after removal) during a precipitation event. We apply the method to ten distinct precipitation events observed in the urban atmosphere of Tokyo, during a 20-day period in the summer of 2014.

## BC-tracer method

**Figure 1:**
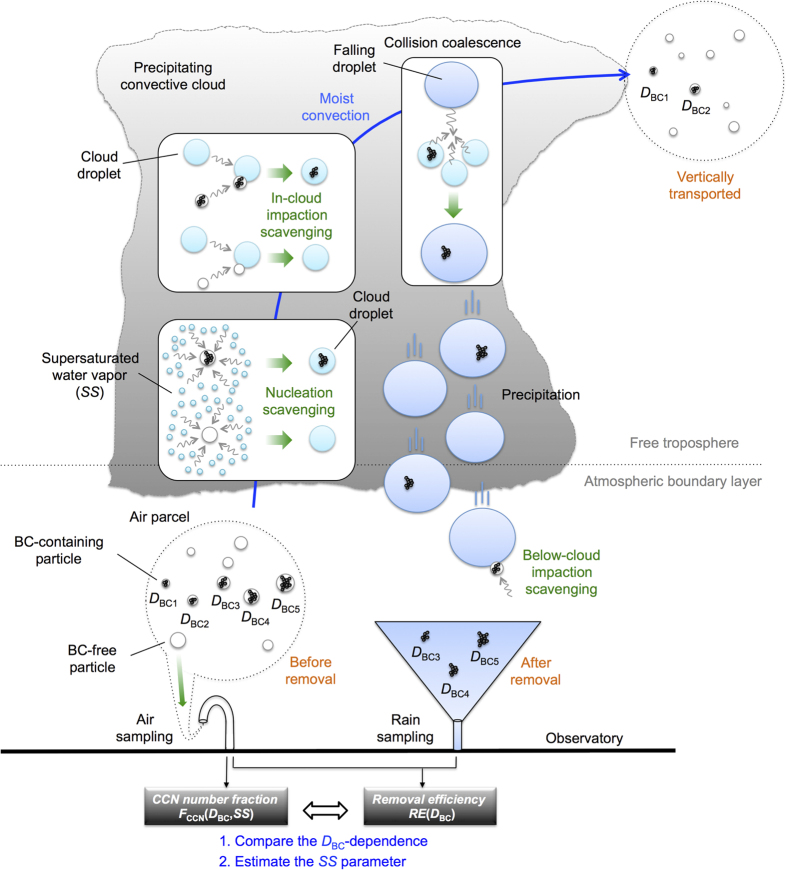
A diagram of the wet removal process for aerosols and our observational method. During the moist convection process, some number fractions of aerosols suspended in an ascending parcel are incorporated into water droplets through nucleation or impaction scavenging and subsequently removed from the atmosphere via precipitation. The mass equivalent diameter *D*_BC_ of each BC-containing particle is invariant throughout the wet removal process. The removal efficiency *RE*(*D*_BC_) is defined as the ratio of the *D*_BC_-resolved number concentrations in rainwater to air. In our method, the observed *D*_BC_ dependences of the CCN fraction *F*_CCN_(*D*_BC_,*SS*) and removal efficiency *RE*(*D*_BC_) are compared. See the text for details.

First, we succinctly explain the key ideas underlying the BC-tracer method. Here, we focus on an air parcel ascending from the atmospheric boundary layer to the free troposphere via the moist convection process (see Fig. 1). Before the ascending parcel is detrained from the precipitating cloud at higher altitudes, some number fractions of aerosols within the ascending parcel are scavenged by below-cloud impaction, nucleation, or in-cloud impaction mechanisms. A particular volume of rainwater collected on the ground can contain scavenged aerosols from many different ascending parcels. Among these aerosols, we focus on BC, a combustion-generated light-absorbing solid carbonaceous aerosol^13^. BC is chemically inert and insoluble in water, and the mass equivalent diameter (*D*_BC_) of each BC particle is invariant in rainwater^14^ (see the Methods section). Therefore, we can observe the *D*_BC_ dependence of the wet removal efficiency via simultaneous ground-based measurements of the *D*_BC_-resolved number concentration of BC in air^16^,^17^ and rainwater^14^,^15^. Namely, we can use an observable invariant, *D*_BC_, as an identifier for each family of particles containing a particular mass of BC and examine whether some families are more susceptible to wet removal than others. The physical properties of BC-containing aerosols (internal mixtures of BC and other aerosol compounds) that affect removal processes, such as particle size and void-free density, are not very dissimilar to those of BC-free aerosols (i.e., sulfates and organics). In addition, BC-containing aerosols serve as CCN in a similar manner to that of BC-free aerosols^18^. Thus, observational results obtained for BC-containing aerosols are applicable to other BC-free aerosols because the physical mechanisms of aerosol scavenging are independent of the aerosol’s internal structure.

Next, we explain the observation and data analysis methods underlying the BC-tracer method. A schematic diagram of the data analysis is shown in Supplementary Fig. S1, and all the symbols used in this study are summarized in Supplementary Table S1. We measure both the *D*_BC_-resolved number concentration of BC-containing aerosols in surface air (*N*_air_(*D*_BC_), units of m^–3^) prior to removal and the *D*_BC_-resolved number concentration of BC particles in rainwater (*N*_rain_(*D*_BC_), units of L^–1^) after removal. *RE*(*D*_BC_) ≡ *N*_rain_(*D*_BC_)/*N*_air_(*D*_BC_) is interpreted as the *D*_BC_-resolved wet removal efficiency of BC-containing aerosols. The *D*_BC_ dependence of *RE*(*D*_BC_) is a direct reflection of the scavenging mechanism experienced by the removed BC-containing aerosols. On the other hand, the magnitude of *RE*(*D*_BC_) can depend on the macroscopic dynamical processes and the degree of evaporation of falling rain droplets. Because our current focus is the aerosol’s scavenging mechanism, only the *D*_BC_ dependence of *RE*(*D*_BC_) is of interest herein. Along with the measurements of *RE*(*D*_BC_), the *D*_BC_-resolved CCN fraction before removal *F*_CCN_(*D*_BC_,*SS*) ≡ *N*_CCN_(*D*_BC_,*SS*)/*N*_air_(*D*_BC_) is estimated using *κ*-Köhler theory^19^ from a measured amount of coating materials on the BC (*R*; the dry shell-to-core ((BC + non-BC coating)/BC) diameter ratio) and the hygroscopicity parameter (*κ*) of non-BC aerosol compounds that likely form a coating on the BC^20^. Here, *N*_CCN_(*D*_BC_,*SS*) is the *D*_BC_-resolved number concentration of BC-containing CCN under a particular supersaturation (*SS*). In this study, the *F*_CCN_(*D*_BC_,*SS*) is evaluated only for 185 nm ≤ *D*_BC_ ≤ 370 nm, for which the *R* data are available. In addition to *F*_CCN_(*D*_BC_,*SS*), we theoretically estimate the *D*_BC_-resolved number fraction of BC-containing aerosols in an ascending parcel that will be scavenged by the impaction with water droplets in cloud (*F*_ic-imp_(*D*_BC_)) and below cloud (*F*_bc-imp_(*D*_BC_)). The detailed procedures for estimating the *F*_CCN_(*D*_BC_,*SS*), *F*_ic-imp_(*D*_BC_), and *F*_bc-imp_(*D*_BC_) are explained in the Methods section. Finally, we compare the calculated *D*_BC_ dependence of *F*_CCN_(*D*_BC_,*SS*), *F*_ic-imp_(*D*_BC_), and *F*_bc-imp_(*D*_BC_) with the observed *D*_BC_ dependence of *RE*(*D*_BC_). In this study, the possible effects of the re-generation of scavenged aerosols by droplet evaporation and any ice processes are not considered in our data analysis.

## Results

We applied the above method to ten distinct precipitation events (Table 1) observed at the Hongo campus of the University of Tokyo during a 20-day period in the summer of 2014. The details of the observation site and sampling methods are explained in the Methods section and in Supplementary Fig. S2. Approximately 30% of the precipitation during the observation period occurred when a cold front associated with a cyclone passed through the observation site. Approximately 30% of the precipitation occurred due to a stationary front sustained over central Japan. Precipitation also occurred due to the influence of a typhoon approaching the main island of Japan. Monitoring of the vertical aerosol distribution using a light detection and ranging (LIDAR) system installed near the observation site revealed that the urban aerosols in the Tokyo metropolitan area were highly concentrated below the cloud base altitude (< ~1 km). In each precipitation event, the horizontal scale of precipitating convective cell typically did not exceed ~10 km, as revealed by the rain radar maps of the Tokyo metropolitan area. For these reasons, we assume that the BC-containing aerosols in the surface air observed within the last 1-h period before the onset of precipitation represent the initial condition of the BC particles observed in rainwater. This 1-h assumption is not critical for the following discussion; the results are similar if we use the data observed within the last 2-h period before the onset of precipitation.

**Figure 2:**
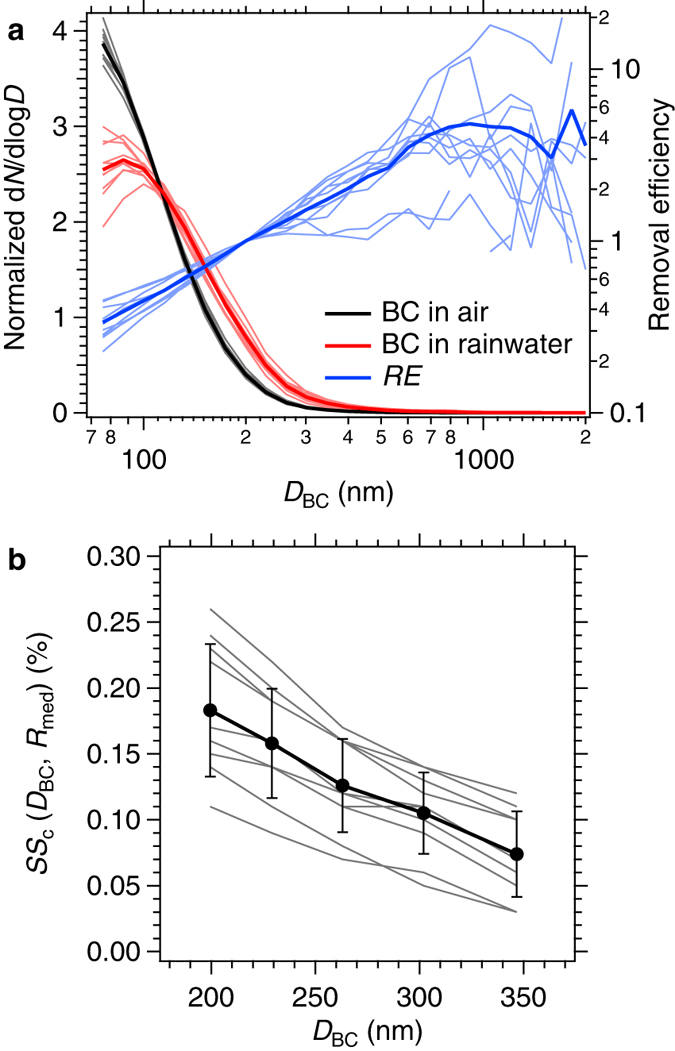
*D*_BC_-resolved microphysical properties of BC-containing aerosols for all precipitation events. (**a**) The number size distribution of BC in air (i.e., the initial distribution) and in rainwater (i.e., the removed distribution) and the resulting removal efficiency (*RE*) for each of 10 precipitation events (thin lines). The size distribution was normalized by the total number concentration. Bold lines show the average result for the 10 events. (**b**) Critical supersaturation (*SS*_c_) of BC-containing aerosols in the air as a function of *D*_BC_ and the median shell-to-core diameter ratio (*R*_med_) for each of the 10 precipitation events (thin lines). The bold line shows the average result for the 10 events. The bars indicate a range of 1 standard deviation.

Figure 2a shows the normalized number size distribution of BC in the air (i.e., the initial distribution) and in rainwater (i.e., the distribution of removed BC) and the resulting removal efficiency *RE*(*D*_BC_) for each of the 10 precipitation events. Each *RE*(*D*_BC_) was scaled to *RE*(200 nm) = 1 for illustrative convenience. The *RE*(*D*_BC_) exhibits a steep increase with *D*_BC_: on average, *RE*(100 nm) ~0.5 and *RE*(400 nm) ~2. The observed *D*_BC_ dependence of *RE*(*D*_BC_) is qualitatively consistent with the observed *D*_BC_-resolved CCN activity of BC particles before removal, as shown in Fig. 2b. Figure 2b shows the *D*_BC_-dependent critical supersaturation *SS*_c_(*D*_BC_, *R*_med_) of BC-containing aerosols for 185 nm ≤ *D*_BC_ ≤ 370 nm for the same events shown in panel (a); *R*_med_ denotes the median *R* value observed for BC-containing aerosols with a particular *D*_BC_. The steep decrease in *SS*_c_(*D*_BC_, *R*_med_) with *D*_BC_ (i.e., higher cloud-nucleating ability for particles with larger *D*_BC_) results from the relatively small amount of coating material on BC (*R*_med_ ~ 1.1) in the size range of 185 nm ≤ *D*_BC_ ≤ 370 nm. For this size range, the observed *R*_med_ values were not strongly dependent on *D*_BC_, and, thus, the total diameter of BC-containing particles (BC + non-BC coating) and the coating mass increased with the increase in *D*_BC_. Therefore, particles with larger *D*_BC_ nucleate cloud droplets more easily, and this tendency is consistent with the *D*_BC_ dependence of *RE*(*D*_BC_) shown in Fig. 2a. The *SS*_c_(200 nm, *R*_med_) value for each precipitation event is listed in Table 1 to show the event-dependent variation of the typical *SS*_c_ value.

**Figure 3:**
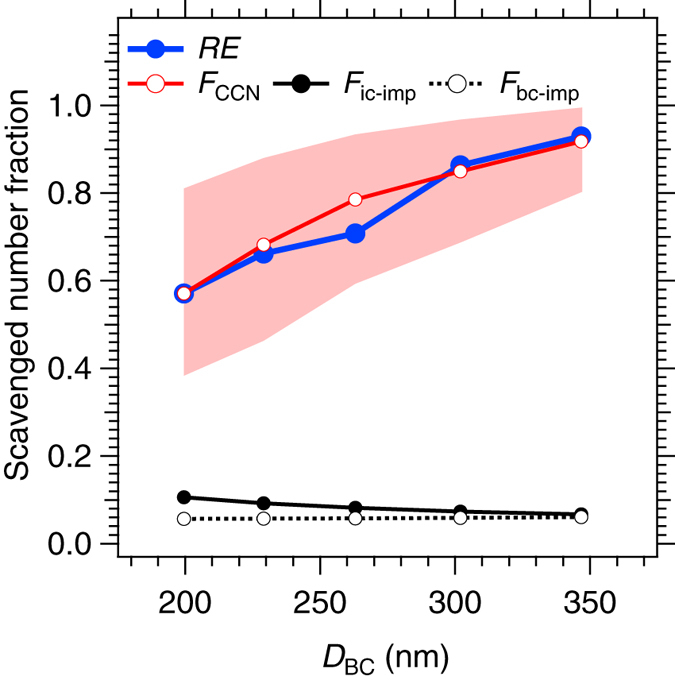
*D*_BC_-resolved wet removal efficiency (*RE*) and the scavenged number fractions (*F*_CCN_, *F*_ic-imp_, and *F*_bc-imp_) of BC-containing aerosols for a selected precipitation event. The results for the event No. 4 are shown. The solid and dashed black lines show the upper limits of the theoretically-estimated number fractions scavenged by the in-cloud impaction *F*_ic-imp_(*D*_BC_) and the below-cloud impaction *F*_bc-imp_(*D*_BC_), respectively (see the Methods section for details). The red line shows the CCN number fraction *F*_CCN_(*D*_BC_,*SS*_est_), where *SS*_est_ denotes the *SS* value that provides the best agreement between *F*_CCN_(*D*_BC_,*SS*) and *RE*(*D*_BC_). The *SS*_est_ value was determined to be 0.25% in this event (No. 4). The blue lines show *RE*(*D*_BC_) scaled to *RE*(200 nm) = *F*_CCN_(200 nm,*SS*_est_). The shading shows the range of *F*_CCN_(*D*_BC_,*SS*) for *SS*_min_ < *SS* < *SS*_max_. Here, the assumed values of *SS*_min_ and *SS*_max_ are 0.18% and 0.38%, respectively.

In each precipitation event, we examined whether the observed *D*_BC_ dependence of the removal efficiency *RE*(*D*_BC_) within the 185 nm ≤ *D*_BC_ ≤ 370 nm range can be quantitatively explained solely by the CCN fraction *F*_CCN_(*D*_BC_,*SS*_est_), where *SS*_est_ denotes the *SS* value that provides the best fit between *RE*(*D*_BC_) and *F*_CCN_(*D*_BC_). Figure 3 shows the *F*_CCN_(*D*_BC_,*SS*_est_) for precipitation event No. 4. Corresponding results for other 6 events are shown in Supplementary Fig. S3. As in this event, in each of other precipitation events, we found good agreement between the *D*_BC_ dependence of *RE*(*D*_BC_) and *F*_CCN_(*D*_BC_,*SS*_est_) for a particular *SS*_est_ value between 0.08% and 0.45% (Table 1). These *SS*_est_ values seem plausible from the theory: the maximum supersaturation predicted by an adiabatic cloud parcel model does not exceed ~1.0% under realistic meteorological conditions (i.e., upward velocity and aerosol concentration)^21^. The average *SS*_est_ for each precipitation type of cold front, stationary front, and typhoon were 0.22%, 0.12%, and 0.30%, respectively. It is important to note that the *SS*_est_ is the effective supersaturation: a weighted average of the maximum supersaturations experienced by BC-containing aerosols in many different ascending air parcels, all of which contribute to the concentration of BC particles in the observed rainwater. The theoretically-estimated upper limits of the *F*_ic-imp_(*D*_BC_) and *F*_bc-imp_(*D*_BC_) values for an ascending air parcel within the range 185 nm ≤ *D*_BC_ ≤ 370 nm are also shown in Fig. 3. Our theoretical estimates reveal that under realistic assumptions, *F*_ic-imp_(*D*_BC_) always shows a monotonic decrease with *D*_BC_, and *F*_bc-imp_(*D*_BC_) shows no appreciable *D*_BC_ dependence (see the Methods section). Therefore, neither in-cloud nor below-cloud impaction scavenging mechanisms can account for the steep increases of *RE*(*D*_BC_) observed in this particle size range. Furthermore, the maximum values of *F*_ic-imp_(*D*_BC_) and *F*_bc-imp_(*D*_BC_) do not exceed 0.11 and 0.06, respectively, both of which are much smaller than *F*_CCN_(*D*_BC_) for this size range.

From the good agreement of the observed *D*_BC_ dependence between *RE*(*D*_BC_) and *F*_CCN_(*D*_BC_, *SS*_est_), along with our theoretical estimates that both *F*_ic-imp_(*D*_BC_) and *F*_bc-imp_(*D*_BC_) are much less than *F*_CCN_(*D*_BC_, *SS*_est_), we conclude that nucleation scavenging is the dominant process via which the BC-containing particles are incorporated into water droplets and irreversibly removed, at least for 185 nm ≤ *D*_BC_ ≤ 370 nm. The chemical composition of an aerosol affects the *SS*_c_ value but does not affect the physics of the scavenging processes. Therefore, this conclusion can be generalized to other BC-free aerosols with a similar particle size.

## Discussion

Our observations using the water-insoluble BC as a particle tracer present conclusive data to prove the hypothesis suggested by previous theoretical^7^,^8^ and observational studies^11^,^12^: nucleation scavenging is predominant over other scavenging mechanisms and therefore controls the wet removal efficiency of accumulation-mode aerosols. This work firmly indicates the importance of refining the parameters that affect the nucleation scavenging process (i.e., the size, composition, and mixing state of aerosols in addition to the maximum supersaturation experienced by the ascending air parcels) for future development of regional/global aerosol models. Such sophistication in models will be required for accurate predictions of the abundance and resulting climate forcings of anthropogenic and natural accumulation mode aerosols. Furthermore, our BC-tracer method provides the supersaturation (*SS*_est_) that effectively controls the typical number fraction of aerosols removed from ascending air parcels. Because the accuracy of the estimate of *SS*_est_ by the present method is higher for steeper slopes in *F*_CCN_-*D*_BC_ correlations, observations in near-source regions where BC particles are relatively fresh and less-coated, such as Tokyo, are more suitable in applying this method. While a supersaturation level was previously estimated from in-situ measurements of aerosol and cloud microphysical properties for a non-precipitating single cloud^22^,^23^,^24^,^25^, our new observational quantity *SS*_est_ is obtained for precipitating cloud clusters, which is the average over space and time. This observational quantity can be compared with the theoretical maximum supersaturation for precipitating cloud clusters, which can be computed using a cloud resolving model^26^. In the future, such observational constraints on the effective supersaturation in precipitating clouds will be invaluable for testing the numerical simulations of aerosol wet removal and vertical transport.

## Methods

### Experimental setup

Our observation site was the Hongo campus of the University of Tokyo (35.71°N, 139.76°E), located within the Tokyo metropolitan area. A major source of BC in Tokyo is reported to be diesel emissions^27^. During the field campaign BC-CARE Tokyo, air samples were aspirated from the sixth floor and rooftop of Science Building 1 (approximately 20 and 40 m above ground level, respectively). Supplementary Figure S2 shows a schematic of the experimental setup. The BC mass and mixing state of individual BC-containing aerosols were measured using a single particle soot photometer (SP2, Droplet Measurement Technology, Boulder, CO, USA) with a laser-induced incandescence technique^16^,^17^,^20^,^28^. The SP2 calibration for BC mass measurement was performed using fullerene soot particles (Stock #40971, Lot #FS12S011, Alfa Aesar, Lancashire, UK) mass-classified with an aerosol particle mass analyser (APM, Kanomax, Osaka, Japan). The mass equivalent diameter of the BC (*D*_BC_) was derived from the BC mass assuming a void-free BC density of 1.8 g cm^–3^. We deployed three different SP2s modified for specific purposes—standard SP2, humidified SP2, and wide-range SP2—as detailed below.

The standard SP2 is able to measure the amount of coating on a BC using a position-sensitive scattering detector in addition to the BC mass. The capability of this SP2 is similar to that of the commercial DMT-SP2 and was fully described in our previous publications^16^,^17^. The detectable BC size range is 70 nm < *D*_BC_ < 850 nm. The amount of coating materials on the BC was estimated by inverting the measured scattering cross section of the BC-containing particles using the light-scattering theory for concentrically stratified spheres. In this inversion, the refractive indices for BC and non-BC coatings were assumed to be 2.26 + 1.26*i* and 1.52 + 0*i*, respectively. This coating analysis was performed for a BC size range of 185 nm < *D*_BC_ < 370 nm.

The humidified SP2 (h-SP2) is able to measure the scattering cross section of the individual BC-containing and BC-free particles at a controlled relative humidity (RH)^20^,^28^. The hygroscopicity parameter κ of non-BC materials for individual BC-containing and BC-free aerosols were estimated based on h-SP2 measurements for mass-sorted aerosol flows. In this system, dried ambient aerosols (RH < 10%) were first mass selected by the APM and then introduced into the h-SP2 at a controlled RH of 85%. The hygroscopicity data obtained by this system during the field campaign were included and fully discussed in our recent publication^20^.

The wide-range SP2 (w-SP2) is able to measure the BC mass in an extended size range of 70 nm < *D*_BC_ < 2000 nm by utilizing a low-sensitivity incandescence detector instead of a position-sensitive scattering detector^14^. In our observations, the w-SP2 was used to measure the *D*_BC_-resolved number concentrations of BC-containing particles in air and rainwater.

Next, we describe our rain sampling system installed on the 12th floor (Supplementary Fig. S2). The size-resolved number concentration of the rain droplets was measured with a laser precipitation monitor (Adolf Thies GmbH & Co. KG, Göttingen, Germany) on the rooftop. During non-precipitation periods (rain rate < ~1 mm h^–1^), the w-SP2 directly sampled the ambient aerosols downstream of a Nafion dryer. During precipitation periods, the rainwater droplets collected by a 30-cm-diameter plastic funnel installed on the rooftop were continually transferred to a small buffer tank (glass bottle) with a volume of ~5 mL. The rainwater in the buffer tank was transferred to a pneumatic nebulizer (Marin-5, Cetac Technologies Inc., Omaha, NE, USA) driven by a continuously operated peristaltic pump. When the liquid level sensor attached to the buffer tank sensed a sufficient amount of water, a compressed purified airflow of 16.7 cm^3^ s^–1^ into the nebulizer drove continuous aerosolization of the rainwater. The BC particles aerosolized from rainwater were then measured using the w-SP2. Our recent work^14^ showed that this Marin-5 + w-SP2 system could measure the size-resolved number concentration of BC particles suspended in rainwater, in the size range of 70 nm < *D*_BC_ < 2000 nm, without size distribution artefacts. The work also showed that the coagulation of BC particles suspended in collected rainwater is negligible during the sample storage for at least approximately one month. Because the water/BC volume ratio in the liquid water should not be very dissimilar between falling rain droplets and collected rainwater, the coagulation of BC particles within a rain droplet should also be negligible, considering the much shorter time scale of cloud-precipitation processes. In addition, the laboratory experiment in this work showed that the incorporation of BC particles into liquid water and the subsequent re-aerosolization of these particles do not change the original size distributions of the BC particles. Based on these experimental facts, we assume that neither cloud-precipitation nor the measurement process changes the size of individual BC particles. Our experiment demonstrated that the loss of BC particles in collected rainwater through the funnel and tubing was no more than 7%, without significant changes in the BC size distribution. The funnel on the rooftop was cleaned with purified water once per day. The number of dry-deposited BC particles on the funnel was estimated to be negligible and did not affect the BC concentration in the rainwater^14^.

### Estimates of *F*_CCN_

The technical assumptions made to estimate the CCN number fraction *F*_CCN_(*D*_BC_, *SS*) in our method were as follows. The distribution of coating amount *R* for the specific *D*_BC_ was derived by inverting the scattering signals measured by the standard SP2 and then converted to the distribution of critical supersaturation *SS*_c_ according to *κ*-Köhler theory. In this study, we assumed that the aerosol compositions of the coating materials on the BC were identical to those of BC-free aerosols. This assumption is supported by the discussion in our recent publication^20^. Thus, the median *κ* value observed for BC-free aerosols with a mass of 7.4 fg was applied in the *κ*-Köhler calculations of *SS*_c_(*D*_BC_, *R*) for BC-containing aerosols with 185 nm < *D*_BC_ < 370 nm. Because of the predominance of freshly emitted BC particles at the observation site^20^, substantial number fractions of BC-containing particles were identified to consist of nearly bare BC particles (*R* < 1.05). Because of the nonspherical shape of BC-containing particles, our inversion of coating amount from scattering signal should suffer from a substantial uncertainty^20^. To mitigate the unknown artefacts of the *R* measurement for thinly coated BC particles, we applied the following assumptions for the number concentration of BC-containing aerosols with *R* < 1.05. We assumed that the *R* value could not be less than 1.001 and the number concentration of BC-containing aerosols linearly decreased with *R* in the domain of 1.001 < *R* < 1.05. For BC-containing aerosols with *R* > 1.05, we found that the *R* distribution was approximated well by a power function. These empirically determined *R* distribution functions were applied to the calculations of *SS*_c_(*D*_BC_,*R*) and thus *F*_CCN_(*D*_BC_,*SS*) for each precipitation event.

According to *κ*-Köhler theory, the *SS*_c_ value of a thinly coated BC particle is highly sensitive to the amount of coating. For instance, the values of *SS*_c_(200 nm, 1.001) and *SS*_c_ (200 nm, 1.01) under a coating κ of 0.19 are 0.82% and 0.45%, respectively. We investigated the sensitivity of *SS*_est_ to the minimum *R*. For every precipitation event, the change in *SS*_est_ between the two assumptions of minimum *R* values of 1.001 and 1.01 was limited to within 0.1%. In this study, *SS*_est_ was estimated assuming a minimum *R* of 1.001 exclusively.

### Estimates of *F*_ic-imp_ and *F*_bc-imp_

Supplementary Figure S4 shows a schematic of the in-cloud and below-cloud impaction scavenging processes of BC-containing aerosols considered for our theoretical calculations of the *F*_ic-imp_ and *F*_bc-imp_. The number fractions of BC-containing aerosols in an ascending air parcel scavenged by impactions with water droplets in cloud *F*_ic-imp_(*D*_BC_) and below cloud *F*_bc-imp_(*D*_BC_) were theoretically estimated based on the following formulation of the scavenging rate^3^:


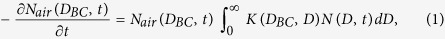


where *N*_air_(*D*_BC_,*t*) is the *D*_BC_-resolved number concentration of interstitial BC-containing aerosols in an air parcel at time *t*, *K*(*D*_BC_,*D*) is the collection kernel for the various attachment processes between a BC-containing particle with a diameter of *D*_BC_ and a water droplet (cloud or rain droplet) with a diameter of *D*, and *N*(*D*,*t*) is the *D*-resolved number concentration of water droplets in an air parcel at time *t*. The number fraction of scavenged BC-containing aerosols via in-cloud (below-cloud) impaction at a specific *t*, *F*_ic(bc)-imp_(*D*_BC_), is then defined as


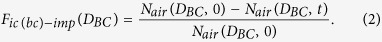


If *N*(*D*,*t*) does not vary with time, *F*_ic(bc)-imp_(*D*_BC_) is determined by


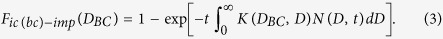


Using Eq. (3), we calculate the upper limits of the *F*_ic-imp_(*D*_BC_) and *F*_bc-imp_(*D*_BC_) under realistic assumptions to estimate the maximum contribution of these impaction scavenging mechanisms to wet removal of BC-containing aerosols in the size range of 185 nm ≤ *D*_BC_ ≤ 370 nm.

In the calculation of *F*_ic-imp_(*D*_BC_), we have taken into account the stochastic collision-coalescence between interstitial BC-containing aerosols and cloud droplets in the ascending air parcel, as well as the collision-coalescence between interstitial BC-containing aerosols and falling rain droplets passing through the air parcel (see Supplementary Fig. S4). The Brownian diffusion, inertial impaction, and interception are considered in the collection kernel *K*(*D*_BC_,*D*)^3^,^29^. To estimate the upper limit of impaction scavenging, we assume that the *N*(*D*,*t*) for cloud and rain droplets are independent of time (altitude) in our calculations. The *N*(*D*) for cloud droplets was assumed to be as follows. The total number concentration of droplets (*N*) was varied from 150 to 1,000 cm^–3^, the size distribution is a lognormal function with a geometric standard deviation of 1.42 and the count median diameter (CMD) was varied from 10 to 20 μm. The *N*(*D*) for falling rain droplets was assumed to be the average size-resolved number concentration of rain droplets observed on the ground. The temperature of the air and water droplets was assumed to be 275 K, and the relative humidity was assumed to be 100%. In our computations, the residence time of the air parcel in the cloud was assumed to be 60 min, which is typical or somewhat longer than the actual residence time. Supplementary Figure S5 shows the calculated *F*_ic-imp_(*D*_BC_) for 185 nm ≤ *D*_BC_ ≤ 370 nm for precipitation event No. 4, which is the same event shown in Fig. 3. The *F*_ic-imp_(*D*_BC_) increases with *N* and CMD. For any *N* and CMD values, the *F*_ic-imp_(*D*_BC_) shows a monotonic decrease with *D*_BC_, as opposed to the observed *D*_BC_ dependence of *RE*(*D*_BC_) (Fig. 3). Even under the extreme assumption resulting in the plausible upper limit of the *F*_ic-imp_(*D*_BC_) (i.e., *N* = 1,000 cm^–3^ and CMD = 20 μm), the *F*_ic-imp_(*D*_BC_) does not exceed 0.11 in the range of 185 nm ≤ *D*_BC_ ≤ 370 nm.

We estimate the *F*_bc-imp_(*D*_BC_) by assuming that the falling rain droplets were constantly passing through the air parcel for 60 min. A plausible upper limit of *F*_bc-imp_(*D*_BC_) was evaluated by doubling the assumed number concentration of rain droplets. In our *F*_bc-imp_(*D*_BC_) calculation, Brownian diffusion, inertial impaction, interception, and thermophoresis were considered for the collection kernel *K*(*D*_BC_,*D*)^3^,^29^. The temperatures of the air and droplets were assumed to be 299 K and 297.5 K, respectively. The relative humidity was set to 90%. The calculated *F*_bc-imp_(*D*_BC_) for precipitation event No. 4 is shown in Supplementary Fig. S5. The *F*_bc-imp_(*D*_BC_) does not exceed 0.06, and there is little *D*_BC_ dependence in the 185 nm ≤ *D*_BC_ ≤ 370 nm range, as opposed to the observed systematic increase of *RE*(*D*_BC_) with *D*_BC_ (Fig. 3).

From these results, we conclude that neither below-cloud nor in-cloud impaction scavenging mechanisms can explain the observed size-dependence of *RE*(*D*_BC_). The same conclusion was obtained for all the precipitation events in this study.

## Additional Information

**How to cite this article**: Ohata, S. *et al*. A key process controlling the wet removal of aerosols: new observational evidence. *Sci. Rep*. **6**, 34113; doi: 10.1038/srep34113 (2016).

## Supplementary Material

Supplementary Information

## Figures and Tables

**Table 1:** Precipitation events observed during the field campaign BC-CARE Tokyo.

Event	Date in 2014	Local time	Precipitation type	Total rain amount (mm)	1-min maximum rain rate (mm h^−1^)	*SS*_c_(200 nm, *R*_med_) (%)	*SS*_est_ (%)
No. 1	27 Jul.	1521–1628	Cold front	4.0	19.7	0.11	0.45
No. 2	9 Aug.	1710–1720	Stationary front	0.9	8.9	0.22	0.19
No. 3	10 Aug.	0002–0445	Typhoon	12.7	15.0	0.14	0.26
No. 4	10 Aug.	1100–1212	Typhoon	12.3	37.4	0.23	0.25
No.5	10 Aug.	1234–1318	Typhoon	9.6	34.2	0.24	0.40
No. 6	10 Aug.	1805–1915	Typhoon	9.2	57.1	0.26	0.30
No. 7	12 Aug.	1626–1632	Cold front	0.2	2.8	0.17	0.09
No. 8	12 Aug.	1720–1810	Cold front	1.0	5.6	0.16	0.12
No. 9	14 Aug.	1327–1643	Stationary front	14.8	27.1	0.15	0.08
No. 10	14 Aug.	1749–1909	Stationary front	2.8	11.2	0.15	0.09
Average	—	—	Cold front	1.7	9.4	0.15	0.22
Average	—	—	Stationary front	6.2	15.7	0.17	0.12
Average			Typhoon	11.0	35.9	0.22	0.30
