# Supplementary material for: A key process controlling the wet removal of aerosols: new observational evidence
Source: Sci Rep. 2016 Oct 5;6:34113. doi: 10.1038/srep34113 (PMC5050421; doi:10.1038/srep34113)
Supplement: Supplementary Information [file srep34113-s1.pdf]

Supplementary information of

"A key process controlling the wet removal of aerosols: new observational evidence"

Sho Ohata[1], Nobuhiro Moteki[1*], Tatsuhiro Mori[1], Makoto Koike[1], and Yutaka Kondo[2]

[1]Department of Earth and Planetary Science, Graduate School of Science, The University of Tokyo, Tokyo, Japan.
[2]National Institute of Polar Research, Tokyo, Japan.

***Corresponding author**

N. Moteki, Department of Earth and Planetary Science, Graduate School of Science, The University of Tokyo, Hongo 7-3-1, Bunkyo-ku, Tokyo, 113-0033, Japan (e-mail: moteki@eps.s.u-tokyo.ac.jp).

Last modified on August 24, 2016

Supplementary Table S1. Symbols used in this paper.

Symbols	Definitions
D_{BC}	Mass equivalent diameter of BC
$F_{bc\text{-}imp}$ (D_{BC})	D_{BC}-resolved number fraction of BC in air that will be scavenged by impaction with rain droplets below cloud
$F_{CCN}(D_{BC},SS)$	D_{BC}-resolved number fraction of BC in air that will be activated as CCN at SS
$F_{ic\text{-}imp}$ (D_{BC})	D_{BC}-resolved number fraction of BC in air that will be scavenged by impaction with cloud or rain droplets in cloud
κ	Hygroscopicity parameter of non-BC aerosols in air
$N_{air}(D_{BC})$	D_{BC}-resolved number concentration of BC in air
$N_{CCN}(D_{BC})$	D_{BC}-resolved number concentration of BC in air that will be activated as CCN at SS
$N_{rain}(D_{BC})$	D_{BC}-resolved number concentration of BC in rainwater
R	Shell-to-core diameter ratio (relative coating amount) of each BC particle in air
$RE(D_{BC})$	D_{BC}-resolved removal efficiency; $N_{rain}(D_{BC})$ / $N_{air}(D_{BC})$
SS	Supersaturation of water vapour
$SS_c(D_{BC},R)$	D_{BC}-resolved critical supersaturation of BC in air
SS_{est}	An SS value providing the best agreement of D_{BC}-dependence between $F_{CCN}(D_{BC})$ and $RE(D_{BC})$

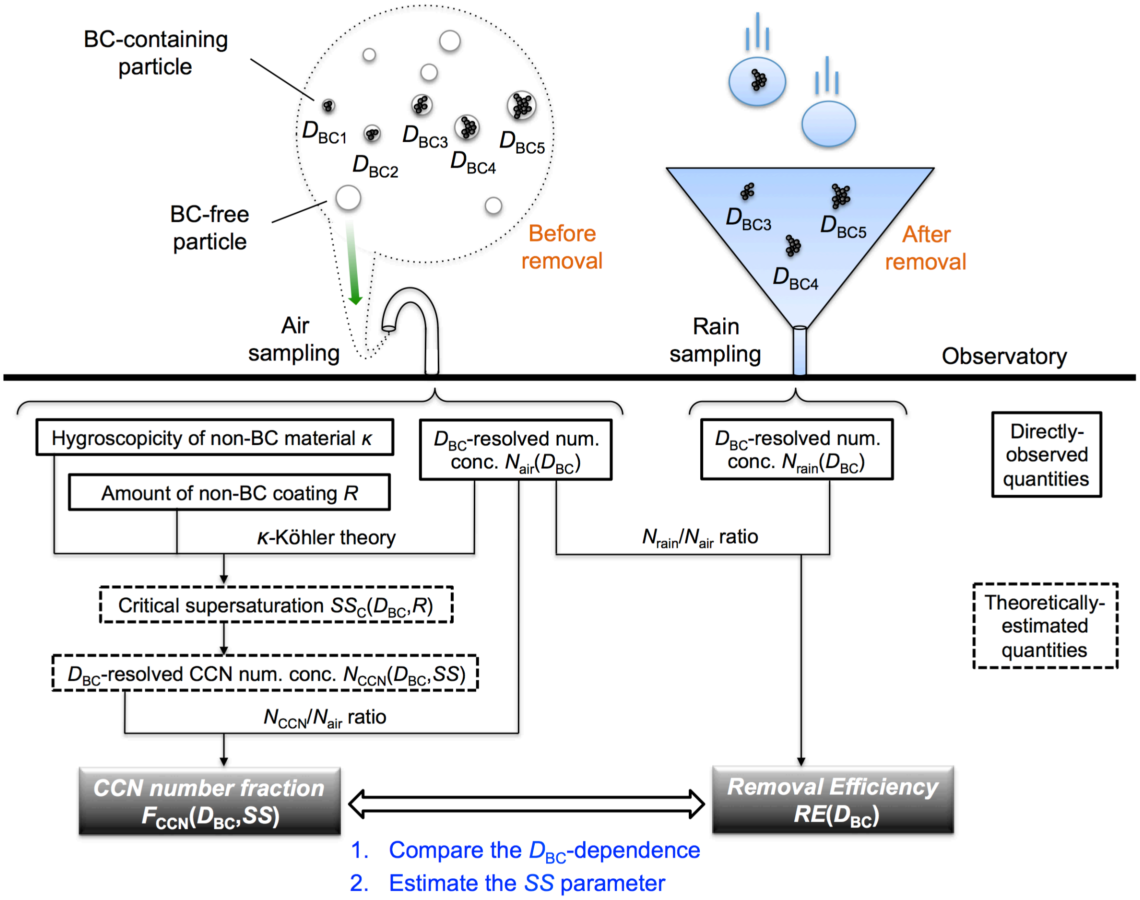

Supplementary Figure S1. A flow chart illustrating the sampling, measurements, and data analysis in our observational method. See the main text for details.

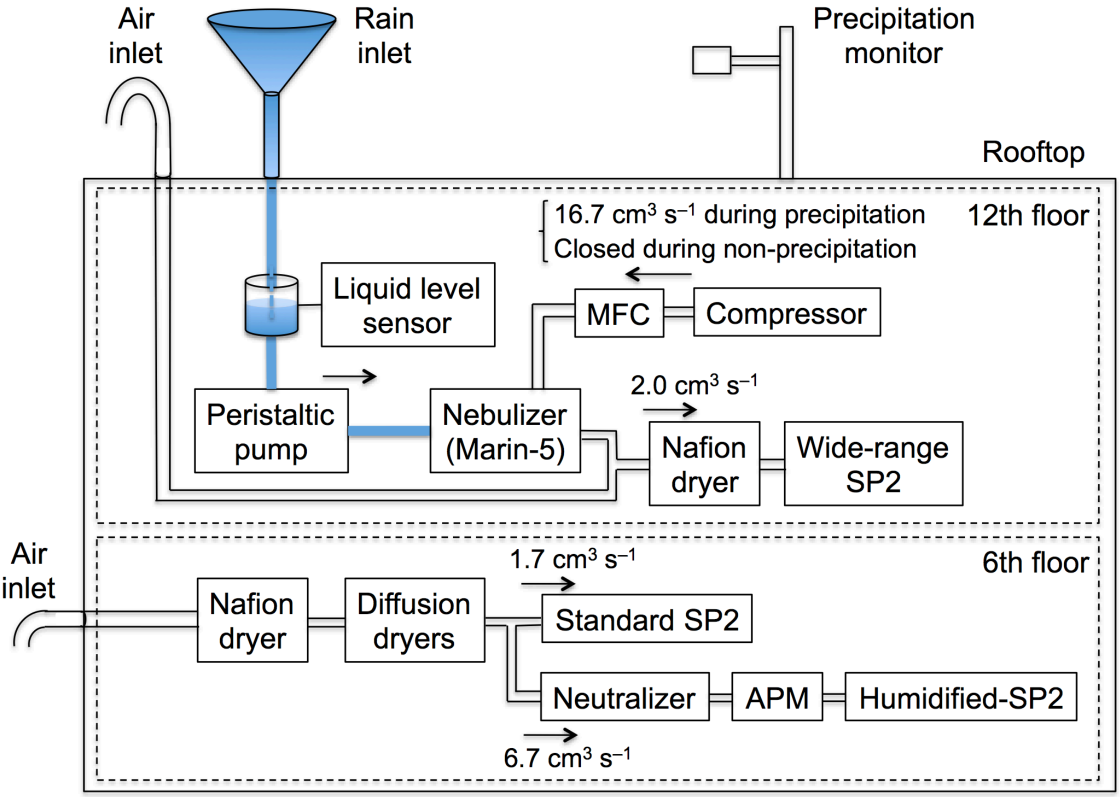

Supplementary Figure S2. Experimental setup. The flow rates are given for room temperature (~298 K) and pressure (~1013 hPa). MFC, mass flow controller; SP2, single particle soot photometer; APM, aerosol particle mass analyser.

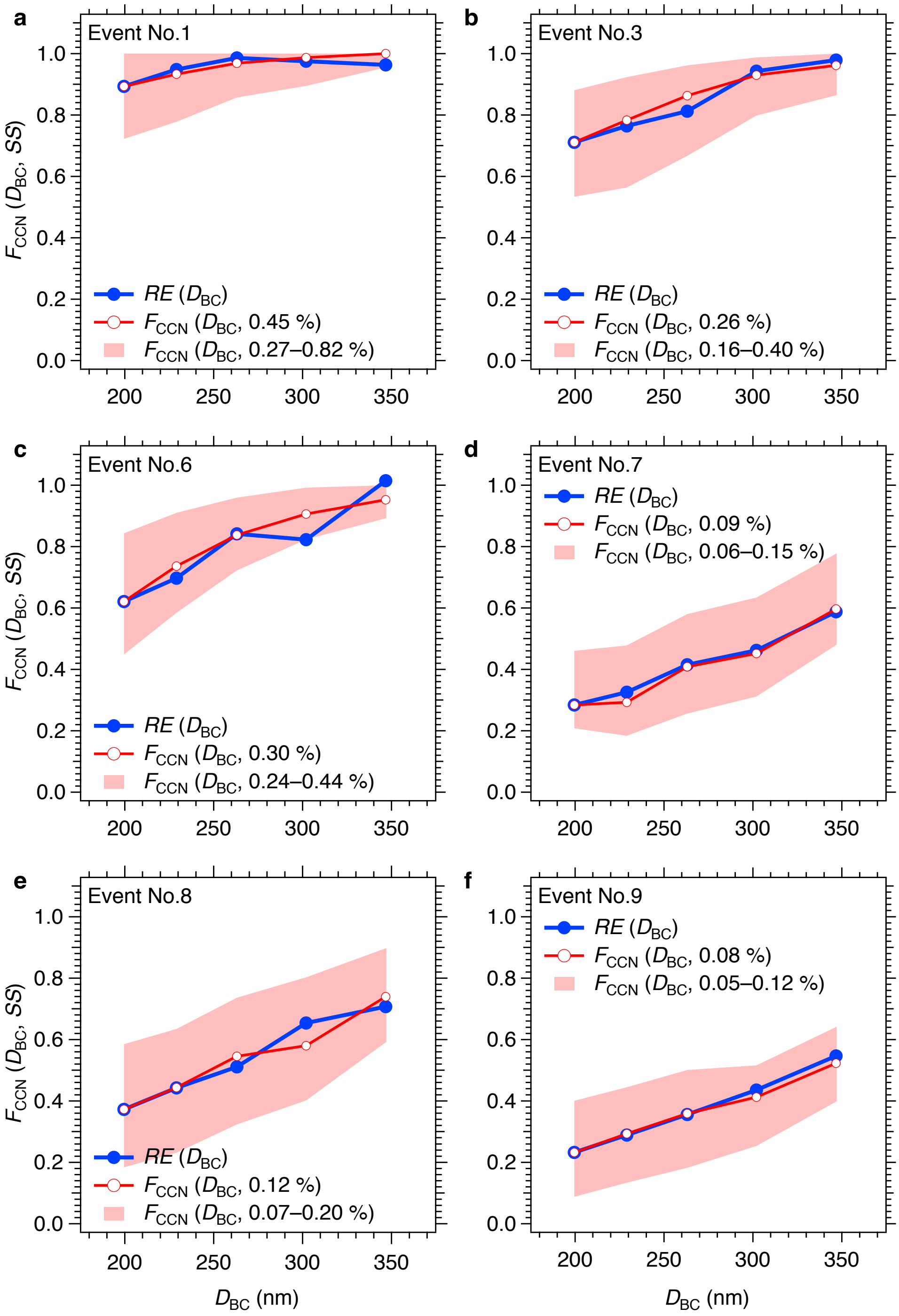

Supplementary Figure S3. D_{BC}-resolved wet removal efficiency (*RE*) and CCN number fraction (F_{CCN}) of BC-containing aerosols for other precipitation events. Same as Fig. 3 in the main text but for the other 6 precipitation events. Since both the estimated $F_{ic\text{-}imp}(D_{BC})$ and $F_{bc\text{-}imp}(D_{BC})$ are much smaller than $F_{CCN}(D_{BC},SS)$ for the size range of D_{BC} = 185–370 nm, they are not shown for illustrative convenience.

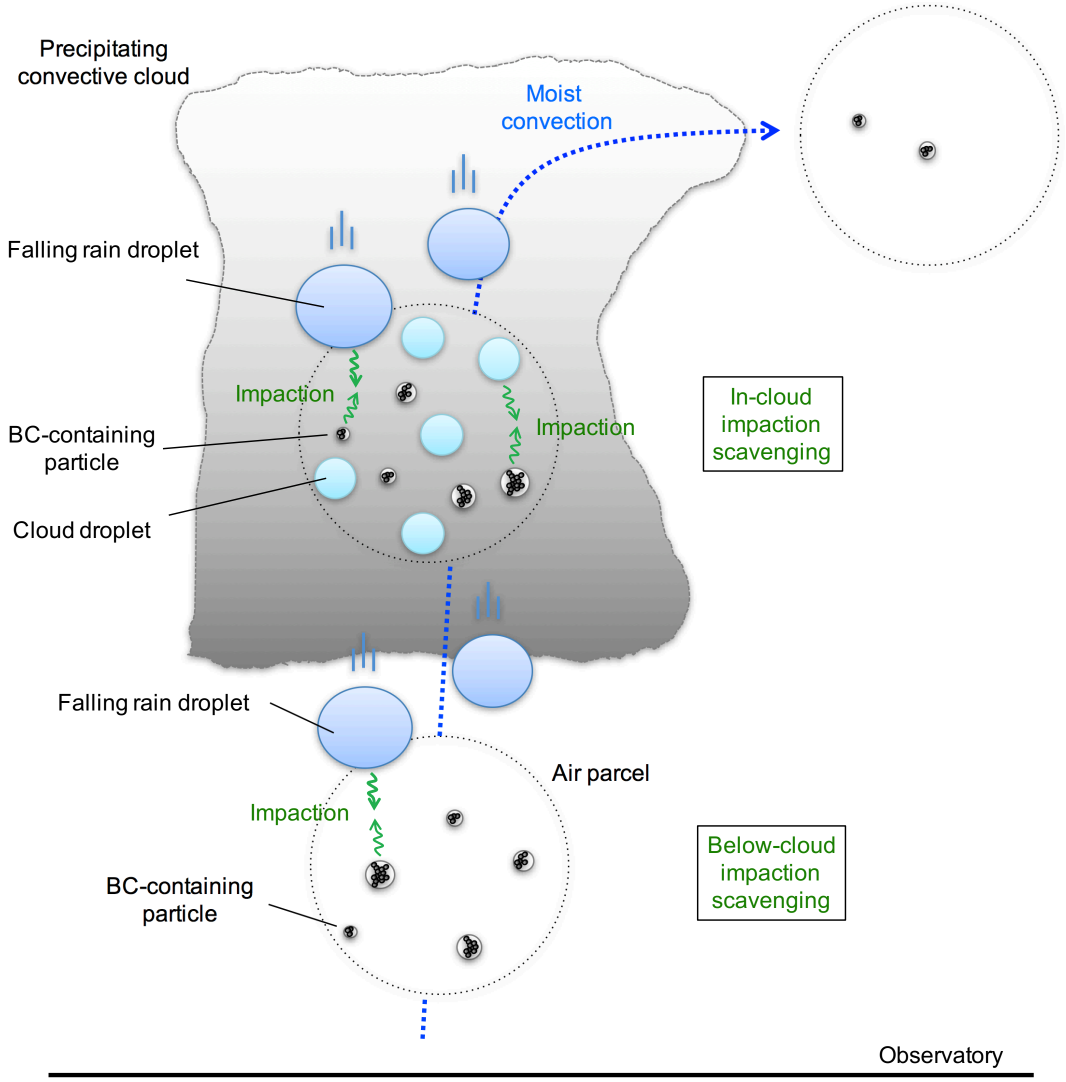

Supplementary Figure S4. A diagram of the in-cloud and below-cloud impaction scavenging mechanisms considered in this study. For the estimates of in-cloud scavenging number fraction $F_{\text{ic-imp}}$, the collision of a BC-containing particle with a cloud droplet and a failing rain droplet is considered. For the estimates of below-cloud scavenging number fraction $F_{\text{bc-imp}}$, the collision of a BC-containing particle with a failing rain droplet is considered. The number size distribution of cloud droplets and the residence time of an air parcel in cloud and below cloud are assumed (see the Methods section).

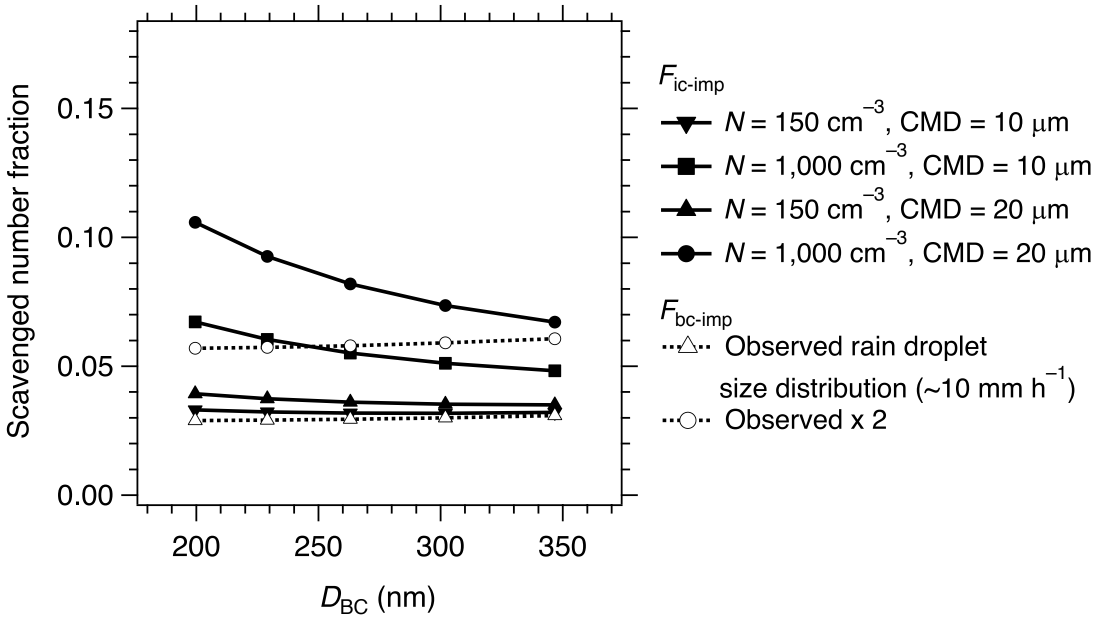

Supplementary Figure S5. D_{BC}-resolved number fraction scavenged by impaction in cloud ($F_{ic\text{-}imp}$) and below cloud ($F_{bc\text{-}imp}$) for a selected precipitation event. The results for the event No. 4 are shown. The solid and dashed lines show the estimated $F_{ic\text{-}imp}$ and $F_{bc\text{-}imp}$, respectively. The different markers indicate various assumptions of the number size distributions of water droplets.
